# Deep questions about the nature of early-life signals: a commentary on Lister (1673) ‘A description of certain stones figured like plants’

**DOI:** 10.1098/rsta.2014.0254

**Published:** 2015-04-13

**Authors:** Martin Brasier

**Affiliations:** Department of Earth Sciences, University of Oxford, South Parks Road, Oxford OX1 3AN, UK

**Keywords:** Martin Lister, palaeobiology, astrobiology, crinoid fossils, biogenicity, taphonomy

## Abstract

In 1673, Martin Lister explored the preservation of ‘St Cuthbert's beads’ plus other fossil crinoid remains from approximately 350 Ma Carboniferous limestone in northern England. He used taphonomic evidence (transport, disarticulation, burial and cementation) to infer an origin as petrified plant remains, in contrast with his views expressed elsewhere that fossil mollusc shells could have formed abiogenically, by ‘plastic forces’ within rock. Lister also observed pentagonal symmetry, now seen as characteristic of living echinoderm skeletons. A postscript from John Ray supports Lister's ‘taphonomic’ observations and accepts the biogenicity of these fossil ‘vegetables’. Ray then concluded with a prophecy, predicting the discovery of comparable living fossils in remote ocean waters. These early discussions compare with current debates about the character of candidate microfossils from the early Earth and Mars. Interesting biomorphs are now tested against the abiogenic null hypotheses, making use of features such as those pioneered by Lister, including evidence for geological context, rules for growth and taphonomy. Advanced techniques now allow us to extend this list of criteria to include the nanoscale mapping of biology-like behaviour patterns plus metabolic pathways. Whereas the science of palaeobiology once began with tests for biogenicity, the same is now true for geobiology and astrobiology. This commentary was written to celebrate the 350th anniversary of the journal *Philosophical Transactions of the Royal Society*.

## 1. Introduction

In this earliest known journal article on the science of palaeontology [1], we observe a great seventeenth century naturalist—Martin Lister—grappling with the nature of fossils. Are these the remains of formerly living organisms (now extinct) or could they have been produced without the need for biology? As explored below, Lister made some crucial and insightful observations about this dilemma, relating to the fields we would now call ‘taphonomy’ and ‘biogenicity criteria’. His observations therefore presage current debates about the earliest signs of life on Earth and Mars [2–7].

**Figure 1.**
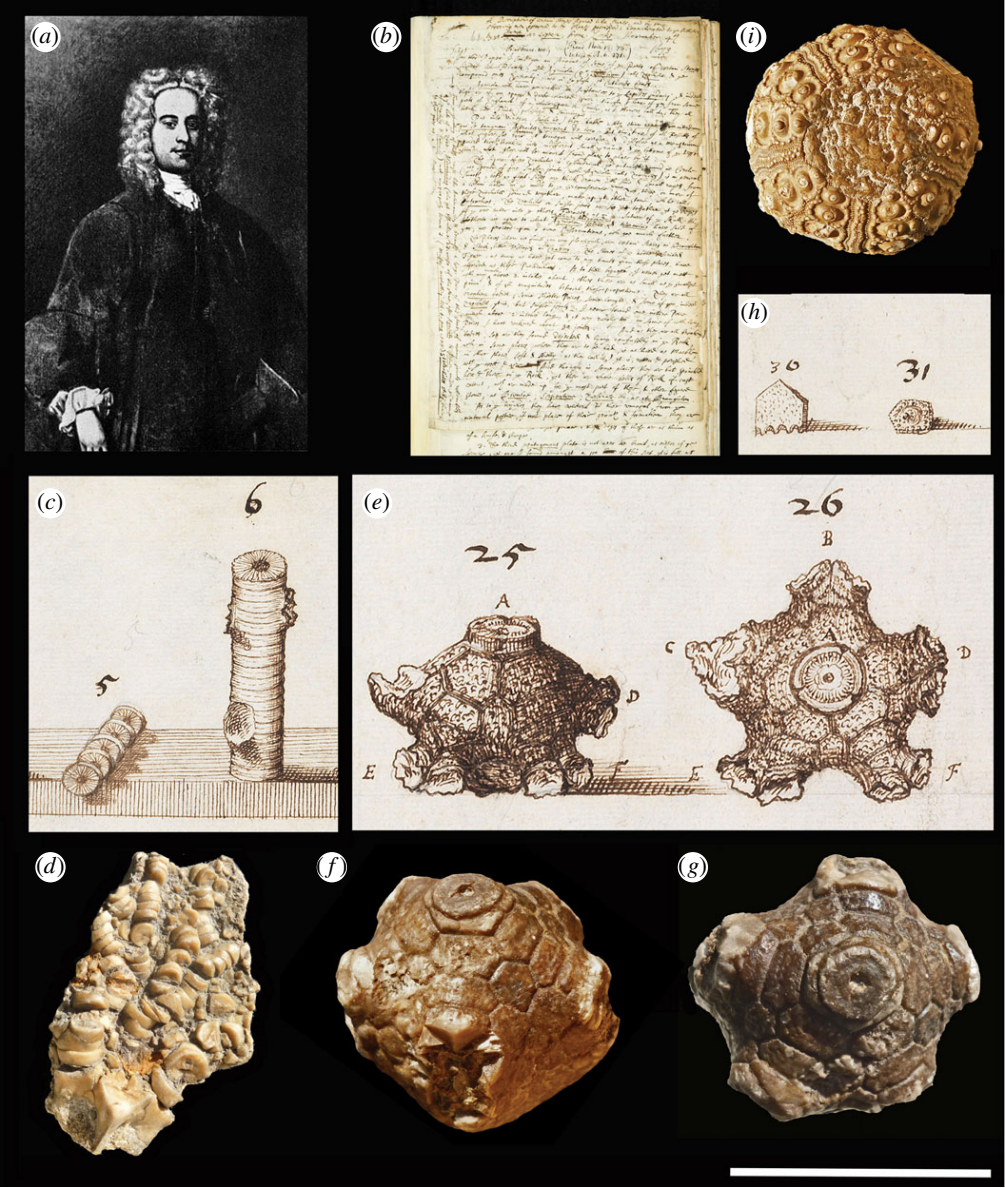
Martin Lister and his ‘plant’ fossils of 1673. (*a*) Portrait of Martin Lister. (*b*) Handwritten first page of Martin Lister's letter, as read before the Royal Society on 13 November 1673. (*c*) Numbered sketches hand drawn by Lister, here showing two fossil ‘rock plants’ (crinoid stems) from the Carboniferous limestone of Yorkshire, UK, with the specimen on the left showing evidence for ‘joynts’ (ossicles) ‘slipped and out of order’. (*d*) Carboniferous limestone fossils showing similar features. (*e*) Numbered sketches by Lister showing lateral and basal views of a ‘radix’ or ‘root’ (actually a fossil crinoid calyx), showing the bases of arm-like features (brachia), rows of polygonal plates around the calyx and a portion of the stem with its central canal. (*e*,*f*) Comparable fossil specimens showing similar features. (*h*) Numbered sketches by Lister showing a ‘pentagonous’ plate (on the left) and another from Northamptonshire, UK, with sculpture resembling an cidariid echinoid interambulcarum (on the right). (*i*) A comparable Jurassic echinoid test, adoral view. (Sources: (*b*,*c*,*e*) Copyright The Royal Society; (*d*,*f*,*g*,*i*) Oxford University teaching collections.) Scale bar, 50 mm (*d*,*f*,*g*,*i*).

Although Lister (figure 1*a*) is now revered as an early expert on both mollusc shells and spiders [8,9], his historically important paper concerns what we would now recognize as the fossil remains of crinoids (‘sea lillies’; figure 1*c*–*g*), a largely extinct class of the phylum Echinodermata that also includes sea urchins and star fish. Interestingly, the animal fossils he puts before us were, at the time of writing, among the oldest known, being some 320–350 Myr old (Lower Carboniferous). None of this was known to Lister, of course, for that took another couple of centuries to emerge.

**Figure 2.**
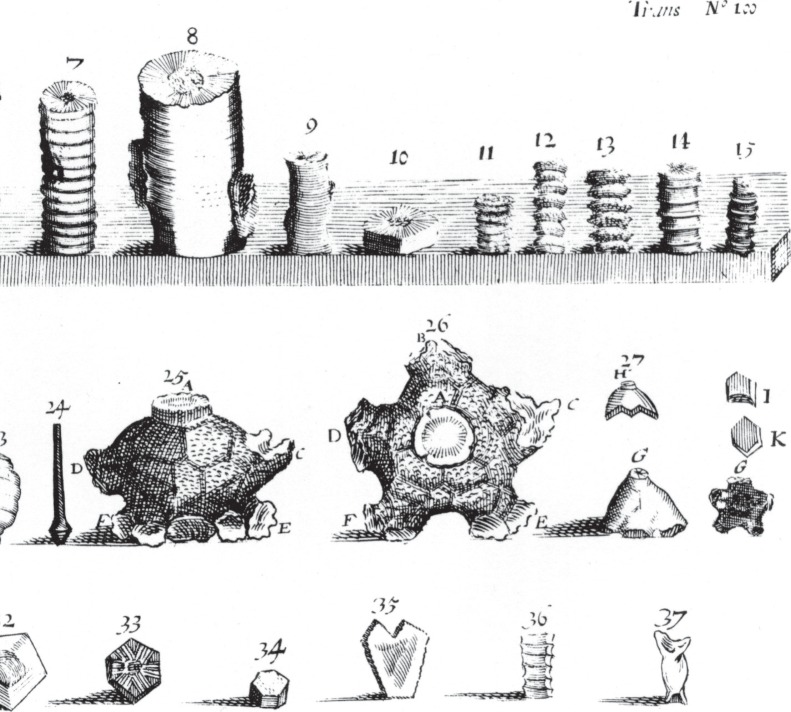
These copper-plate engravings appeared in Lister (1673) as figs. 1–37 of his ‘Tab. 1’ [1]. They are reversed from his original drawings. Loosely translating his descriptions [1], to make palaeontological meanings clearer, these show the following: 1, a single ‘joynt’ with very fine and small rays; 2, ‘joynt’ with ‘pith’ [central canal] bored through in the manner of a cinquefoil; 3, single oval ‘joynt’ with scarcely visible rays, and a small point in place of the ‘pith’; 4, single ‘joynt’ with a very large ‘pith’; 5, a pack of dislocated ‘joynts’ kept in the correct order; 6, a very long column having many smooth ‘joynts’ with the branches broken off; 7, a column with smooth ‘joynts’ and without branches; 8, the biggest column, with stumps of branches; 9, a smooth column with very smooth and numerous ‘joynts’; 10, one of the widest and most deeply ‘joynted’ pieces of a column; 11, a column with numerous poorly ordered knot-like ornaments; 12, a column with only a single row of ‘knots’ in the centre of each ‘joynt’; 13, a column with three rows of ‘knots’ on each ‘joynt’; 14, a smooth column, with each ‘joynt’ bearing a single large ridge around the middle; 15, ‘joynts’ that are alternately raised and depressed; 16, a double facet on the edge of each ‘joynt’; 17, alternate ‘joynts’ bearing edged facets; 18–20, certain other differences noted in the paper, but unclear in the engravings; 21, a column bearing a distinct side branch; 22, a branch broken off from a column; 23, a column shaped like a fruit; 24, a ‘sastigium or summitas’ [possible echinoid spine]; 25, a root-like ‘radix’ [crinoid calyx] in lateral view: A shows a ‘joynt’ remaining ‘whence an Entrochos [column] was broken off’; C, E, F, D, show four of the double ‘feet’ [brachia], the rest being hidden from view; 26, the same ‘radix’, seen in plan view: A shows the broken off column; C, B, D, E, F show the five double ‘feet’ [brachia]; note also the hexangular plates with roughened ornament, ‘which incrustrate the stone or cover it all over’; 27, a smaller ‘radix’ [calyx] with smooth plates and five single ‘feet’ [brachia]: H, the top stone; I, one of the five ‘feet’ [brachia]; K, one of the five angular plates which ‘incrustate the middle of the stone’; G, the base; also the same stone seen from the side; G, the same with the hollow bottom facing upwards. The following figures are of plates that are supposed to ‘incrustate divers roots’ [i.e. plates of the crinoid calyx]: 28, a ‘pentagonous’ plate knotted; 29, a thin-edged, smooth ‘pentaganous’ plate; 30, an indented ‘pentagonous’ plate; 31, the Northamptonshire ‘pentagonous plate’ [possible echinoid interambulacral plate from the Jurassic]; 32, a large ‘pentagonous’ smooth plate; 33, a ‘hexagonous’ plate sculpted with ‘angles’; 34, a ‘hexagonous’ plate ‘as deep as broad’; 35, 37, oddly shaped plates; 36, a quadrangular plate, ribbed and indented. (Copyright The Royal Society.)

The title of Lister's paper clearly sets out a major paradox: ‘A description of certain *stones figured* like plants, and by some observing men esteemed to be *plants petrified*’ (my italics). The puzzle concerns the existence within limestone rock of surprisingly complex disc-shaped ‘fossils’, called St Cuthbert's beads (figure 1*c*–*g*). This name reflects not only the presence of a central hole in each disc but also their occurrence around sacred Northumbrian sites. Using his printed words alone, we can readily sketch out the complex morphology he describes. We could even identify some of his fossils with precision. But palaeontology also requires graphic evidence, which Lister provides with an abundance of anatomical drawings (figure 1), turned into copper-plate engravings (figure 2) by ‘my very good friend, Mr William Lodge’ (see [10]). These detailed illustrations were able to demonstrate, for the first time, the range of variation to be found within a single fossil group. The puzzle, however, was to ascertain how they were formed.

## 2. The figured stones hypothesis

**Figure 3.**
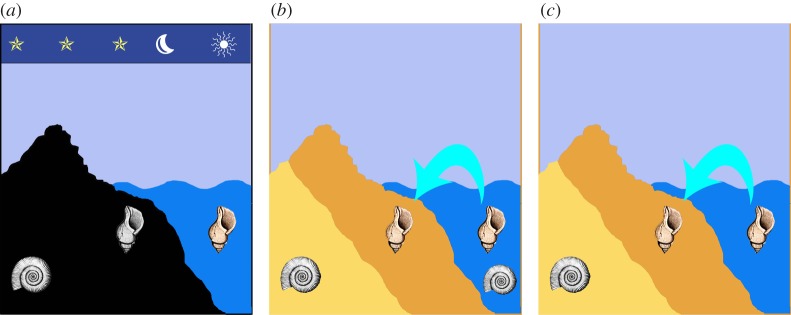
Three alternative hypotheses available for the interpretation of fossils in 1673. (*a*) The figured stones hypothesis of Kircher [13]: fossils found in rocks grow under the influence of ‘plastic forces’, perhaps including cosmic influences; living invertebrates and algae may also grow by spontaneous generation. This view was preferred by Lister for his fossil molluscs [14–16]. (*b*) The biogenic hypothesis of Steno [17–19] and Hooke [20]: fossils found in stratified sediments are explicable as the remains of once living organisms; unfamiliar groups (e.g. ammonites, crinoids) may yet be discovered in remote areas or the deeper ocean. This view was hinted at by Lister [1] and explicitly suggested by Ray [21]. (*c*) The biogenic–extinction hypothesis is similar to (*b*), except that unfamiliar fossils may now be explained by their extinction [22]. This possibility was actually mentioned by Lister [16] for some of his fossil molluscs. The subsequent addition of evolution [23] then brought thinking towards its modern stance.

We need to remember that the word ‘fossil’ comes from the latin word *fossa* (a ditch, or hole). ‘Fossil’ was therefore available for any noteworthy object dug from the ground, meaning that it could be mineral, antiquarian or biological in origin. More importantly, there were two contrasting views about what we now call ‘fossils’ at this time [8–12]. The first was of growth within the Earth as ‘stones figured like plants or animals’ (as stated in Lister's title), produced as the outcome of enigmatic ‘plastic forces’ (figure 3*a*). A second major perspective saw fossils as relics of previously living forms left behind by major changes in sea level (figure 3*b*,*c*).

The concept of fossils as in-between beings—halfway between the living world and minerals—is arguably very ancient, traceable back through the Neo-Platonists of ancient Greece, and perhaps to the Stone Age. Following the establishment of the Christian church in the fourth century AD, plastic forces were more likely to be seen as the work of God the creator, holy men (miracles) or demons (spells). The popular name of ‘St Cuthbert's beads’ for the fossils described by Lister [1] likely reflects such a mode of thought, in which holy beads were transformed into rock. We can still encounter similar concepts today, in religion (e.g. Holy Communion), literature (e.g. Harry Potter) and computer games (e.g. *Mortal Kombat*). Expectations of ‘shape-shifting’ and transformation seemingly lie deep within the human brain.

During the seventeenth century, the plasticity of boundaries was being seriously explored, leading some to test whether ‘fool's gold’ (pyrite, iron sulfide) could be turned into metallic gold [12]. Interestingly, Martin Lister undertook experiments with pyrite [24] and was fascinated by its role in the formation of fossils [8]. Importantly for our story, the boundaries between inert minerals and living matter were also thought to be porous. Thus, when Conrad Gesner, the Swiss naturalist and bibliographer, first illustrated fossil shells alongside modern shells in 1565, a popular inference was that both kinds could generate spontaneously within water or within rock [11,25]. This view was also supported by the eminent naturalist Athanasius Kircher [13], who regarded corals (a group we now regard as simple animals) as transitional between vegetable and mineral because they resembled flowers in shape but had mineral skeletons.

In 1665, an important new line of evidence was provided by microscopic analysis, highlighted by Robert Hooke [20] in *Micrographia*. But even here, Hooke felt free to speculate on the spontaneous generation of plant and animal life from inorganic matter within a water butt [20,26]. Indeed, it took centuries for the concept of spontaneous generation to retreat towards the origins of life alone.

## 3. Towards the biogenic hypothesis

The biogenic hypothesis has deep roots. Greek philosophers, including Strabo, had noted seashells far inland and inferred that such areas had once been covered by the sea [12,26]. A later shift towards biblical thinking encouraged their interpretation as doomed relics of a pre-Flood world (literally ‘antediluvian’) or as remains left behind by the Flood itself (‘diluvial’). The expectation of stories within rocks therefore became one of an act of Creation, followed by decline and decay [12].

These early hypotheses were seriously challenged by the Danish anatomist Nicolaus Steno in 1669, within his book *De Solido* [17]. An English translation was published soon after by the Royal Society [18]. Travelling across Europe, Steno had observed layers of rock containing fossil remains, allowing him to conclude that the history of the natural world could be read layer by layer, with the oldest rocks at the bottom and the youngest at the top (‘the law of superposition’). He argued that his rocks were once aquatic sediments and, most importantly, that many fossils were the remains of plants and animals (figure 3*b*). In so doing, Steno provided the first great challenge to the figured stones hypothesis, noting that fossils did not resemble simple crystals but took on more complex shapes. Those near to each other seemed to be broken like shells on a beach, and now seemed to be in the process of disintegration, not of formation. Most famously, he also examined tongue-shaped fossils (‘glossopetrae’) and compared them with the teeth of modern sharks [19]. He thereby showed that ‘comparative anatomy’ with living forms could help to infer a biological origin for fossil forms.

Back in England, similar conclusions had already been reached by Robert Hooke in 1665, within the pages of *Micrographia* [20]. While much of Hooke's work on fossils and fossilization was only published posthumously [27], *Micrographia* contains a very important comparison between the cellular fabric of living and fossil wood, and also makes plausible suggestions as to how they might have become petrified [20,26,28].

This article by Lister [1] therefore needs to be seen in the context of the ‘biogenic’ hypothesis of Hooke [20] and Steno [17–19]. Other ‘biogenic’ supporters within the Royal Society included Robert Boyle [29] and John Ray ([21,30]; see also [8,12]). Both Ray and Lister had collaborated with Steno while at Montpellier in France in 1668. But while Ray had become an advocate for the ‘biogenic’ origin of fossils, Lister is now seen as either a confirmed sceptic [12] or as sitting on the fence, arguing that ‘fossils were not always the remains of living creatures’ [8]. As explored below, Lister's critical stance was arguably a prudent one for the time and remains so for modern debates about signals remote in time and space.

## 4. The crinoid paper by Martin Lister

We need to appreciate the huge list of unknowns for Lister and his colleagues in 1673. There was no accepted terminology for the chemical elements and minerals of which fossil remains were made. There was no agreement about the nature of sedimentary rocks, or the laws of stratigraphic succession. Nor was there a clear understanding about distinctions between plants and animals. No knowledge was available concerning the morphology of modern crinoids, and hence no appropriate terminology was available for use. And there was certainly no understanding about the phenomena of either extinction or evolution. Lister was therefore faced with trying to decode the remains of organisms (in this case, crinoids) wholly unknown to science as living organisms.

Despite these problems, the structure of Lister's paper seems remarkably modern. The spellings, which can sometimes appear charming and idiosyncratic to modern eyes (e.g. ‘joynts’ for joints), belong to a time before those spellings were settled [31] and at least remain phonetic and recognizable. The paper might even pass today as the first draft of a research project written by a promising student. Using modern terminology, we can also divide Lister's paper under the following headings: previous work; mineralogy and chemical composition; morphological terms used; localities visited; fossil size distribution; presence of natural fossil assemblages; evidence of growth, death and transport in fossils; species description and variation; numbered illustrations and figure descriptions. One might complain, a bit unfairly of course, that there is no geological map or stratigraphic column (the stratigraphic map was a later Lister invention [32]). It might also be criticized for being written in the first person, with an Almost random Scatter of capital Letters and italicized *words*, and then printed with elongated ‘f-like esses’. For a paper upon ‘fossils’, this can seem just a bit distracting, though all of this was completely normal for the time.

Fortunately, his descriptive features plus 37 illustrations (figure 2) clearly allow us to identify his fossils, gathered from the Craven district of Yorkshire, UK, as the calcitic remains of crinoid skeletons. He uses the term ‘Trochite’ for what we would now call a crinoid ossicle (figure 2, items 1–4), and the term ‘Entrochites’ or ‘Trochitae’ for collections of crinoid ossicles including what we would call columnals (from the stalk) and brachials (from the arms or brachia; see figure 2, items 5–21). Their calcareous composition he then demonstrates by their vigorous reaction to acetic acid (‘vinegar’; p. 6182, lines 1–5).

The bulk of the paper provides a detailed description of features (pp. 6184–6191) that were also illustrated (figure 2). Lister carefully assembles both his descriptions and images to show features of the curious, disc-shaped ‘joynts’ (ossicles) that characterized his fossils. Each had a central hole (‘hollows’ or ‘piths’), which in some was pentagonal (‘Cinquefoil’). They formed clusters, showing intervening sutures whose exterior profiles were smooth or ‘indented’. Adjacent ossicles could differ in size, shape and sculpture, forming complex alternating patterns, or be tapering from one end to the other. The outer surfaces of these ossicles could also be smooth, or sculpted by a single row of tubercles (‘knots in a circle’), or several rows of different sizes (‘this is huge pretty’), or be randomly placed. ‘Knots’ could also be replaced by a central ridge (‘joint rise’) or groove (‘double edge’) or a central swelling (‘swelled’). The ‘terminating’ junctions between ossicles were found to be smooth or sculpted by radiating ‘rayes’. While the bulk of these fossils can be confidently identified as crinoids, a few appear to be echnoid fossils, including a cidariid spine (p. 6184, lines 34–38; see figure 2, item 24) plus an interambulacral plate provided with a sculpted boss (p. 6188, lines 11–20; see figures 1*h*,*i* and 2, item 31), both from the Jurassic of Northamptonshire, UK.

More complex fossils included ossicles (‘joynts’) arranged in columns that he found to ‘branch’ from a main ‘stemm’, with diameters getting progressively smaller as the branching proceeds (p. 6185, lines 1–13). By this stage in the paper, Lister was arguably thinking of a large and complex structure that seemingly had distinct rules for growth. Stranger still were his specimens of a crinoid calyx (figures 1*e* and 2, items 25–27), now known to house the main organs of the animal. Unlike Lister, we know that each calyx lies on top of the stalk and bears the branching brachials that caught the food. Lister describes his calices as having ‘the bigness of Walnuts’ and compares them with ‘a Pine Apple or Cone’. He correctly observes and clearly illustrates their construction from calcareous plates (figure 1*e*; cf. figure 1*f*,*g*), which encouraged him to infer that they were parts of a single ‘plant’—connected to the stalks and branches—though he incorrectly suggested they were ‘incrustations of roots’ that had become broken away (p. 6186).

It is especially interesting to observe Lister with respect to the rules for growth. He notes the ‘Cinquefoil’ pattern of the central canal in some stalk ossicles (fig. 2.2; p. 6186, lines 7–8). Some isolated pentameral plates he called ‘pentagonous’ (fig. 2.28–31; p. 6187, line 30 onward; p. 6188, line 1 onward.). He also noted a double row of five plates arranged around the calyx (figure 1*e*; fig. 2.26; p. 6186, lines 36–39), each plate being hexangular (p. 6187, line 1); and brachials in the form of ‘five single feet [arising] at equal distances’ around the calyx (p. 6186, lines 36–37). Although pentameral symmetry has since been regarded as a characterisitic of the echinoderm phylum, it can also be found in plants. Together with the lily-like shape of crinoids, this may have encouraged him to refer to them as ‘plants’.

## 5. Lister's biogenic dilemma

Lister [1] never mentions the work of Nicolaus Steno [17–19] nor does he mention the biogenic hypothesis directly here. This is curious, given that Lister is usually seen as a critic who questioned Steno elsewhere [9,12]. Throughout the paper, indeed, we gain rather the opposite impression—that Lister is pointing towards evidence for life, death, transport and burial. If this is a correct interpretation, then it could be argued that Lister was contemplating the significance of death and burial processes in the fossil record—the discipline called ‘taphonomy’ (e.g. [33]). For example, when reviewing the size and arrangement of ossicles, he makes observations that suggest they have been broken apart long ago:

They are all *broken bodies*; some shorter Pieces some longer, and some of them, indeed, *Trochites*, that is, but single joints…. And as they are all broken bodies, so are they found *dejected* and lying confusedly in the Rock, which in some places, where they are to be had, is as hard as Marble…. (p. 6182, lines 31–38, his italics)

Turning over a page, we see Lister making use of words that suggest he conceived of the fossils as having once been growing, provided with a living posture, then suffering injury and being transported and dislocated at the place of burial:

As to the injuries they have received in the removal from the natural *posture*, if not place of their growth, and formation, they are manifest. For, besides their being all broken bodies, we find many of them depressed and crushed…Again these stones…are many of them strangely dislocated; sometimes two, three, or more of the joynts in a Piece are slipped and out of order or rank, and sometimes a whole *series* of joynts, as when as pack of Crown pieces leans obliquely upon a Table. (p. 6183, lines 6–16; see also fig. 2.5)

To this he then adds a picture of the structures becoming twisted and suffering ‘injuries’:

Further, others I have that are twisted like a Cord, if this possibly may be reckoned amongst the injuries. (p. 6183, lines 16–18)

And later, he even gives us a tantalizing picture of life posture, death and transport:

Some…sare yet visible in their natural place and posture in the described stones: But I find the greatest part of them broken up and heaped together in great confusion in the Rocks. (p. 6187, lines 21–24)

Then, there come seminal observations suggesting that these fossils may have been buried in sediment, and then cemented like a layer of bricks or by sparry calcite:

Lastly, some have their joynts [ossicles], indeed, even in file [columnals], but are yet stuffed with a forrain [foreign] matter, as when bricks are layed in morter. (p. 6183, lines 18–20)

…the substance that covers them (if not the Stones themselves) is Sparr…. (p. 6186, lines 17–18)

An important conclusion here is that these could be different ‘species’ of plants:

there are many other differences to be noted…. Very probably because they are Parts or Pieces of different *Species* of *rock-Plants*. (p. 6184, lines 2–4, his italics)

One interesting conclusion, therefore, is that Martin Lister thought of these as fossilized plants (which, for him, may have included simple animals like corals) which had once lived on the rocks. There is an alternative view, however: that by ‘rock-Plant’, he actually meant a fantastic kind of plant that could grow within the rocks by a process of spontaneous generation [8]. While he leaves it moot, Lister was clearly faced with a dilemma. Of the five-rayed central canal seen in some ossicles, for example, he reveals his puzzlement:

This is most surprising and I know not of any Vegetable, whose Pith is perforate in such a manner. (p. 6186, lines 10–12)

## 6. John Ray and living fossils

The deeper meaning of these fossils was further hinted at in a short postscript by John Ray [21]. This naturalist had collected similar fossil remains from Lindisfarne, and he may well have encouraged Lister to prepare a personal report [8]. Here, Ray recalls his earlier travels to Malta (in the steps of Steno), where he saw branched and stick-like fossils that resembled rock plants (possibly *Thalassinoides* crustacean burrows from the Miocene). Ray then points out the need to understand the rules for growth within living (and, by implication, fossil) plants, through the analysis of both branching and taper. Importantly, he reinforces Lister's taphonomic model, with evidence for breakage, stating that ‘Those Roots [calices and brachials], that you have observed, are a good argument, that these Stones were originally pieces of Vegetables’ [21]. Equally prophetic was his final section, in which he wonders whether such organisms might be found living attached to rocks on the seafloor today, predicting that they might yet be trawled up by fisherman [21].

Ray was therefore speculating about what we would now call ‘living fossils’ (figure 3*b*). Final confirmation of stalked crinoids—living fossils—in deep waters had to wait for almost another century [34].

## 7. Towards biogenicity criteria

Piecing together the observations and interpretations made by Martin Lister [1] and John Ray [21], we can now highlight four basic rules for the biogenicity of candidate fossils which date right back to the very start of the discipline. Such tests still remain important for calibration of the fossil record. They are especially required at the start of a palaeontological field, when the rules can otherwise seem unclear, as in the search for life's origins in the early rock record (e.g. [3,4,26,35]). Much hangs on this, because fossils tend to become increasingly scarce as we dig ever deeper into the past. A single reliable fossil from deep time can therefore wholly transform our understanding of biosphere history.

### (a) An appropriate null hypothesis

This rule comes first because it allows palaeobiologists to set up a hypothesis which will prevail until proved false (loosely adapting the framework of Karl Popper; see [36]). Any newsworthy, and culturally challenging, interpretation must therefore be tested against a less exciting interpretation. This ‘null hypothesis’ is usually regarded as the ‘most boring explanation’. It is boring precisely because it is thought to have a higher probability of being correct. As the Royal Society motto still says: ‘take nobody's word for it’.

For Martin Lister in 1673, the null hypothesis was that his fossils formed by abiogenic, plastic forces within rock [8], as caricatured in figure 3*a*. This was also the view of Kircher [13] and it prevailed for some decades afterwards, following the ideas of Oxford ‘chymist’ Robert Plot [8,37]. While Lister's choice of words shows that he was willing to play with the concept of petrified plants, he also found it much harder to accept the idea of fossilized animals. Elswhere, Lister [38] noted that the character and mineralogy of fossil shells differed from living forms, and also reflected the character of individual quarries, both correct observations in themselves. Suspecting plastic forces at work, he tried growing mollusc shells within beakers of mineral solution mixed with the body juices of molluscs [8,12]. His preferred conclusion about fossil molluscs, therefore, was that variations in size and state of preservation were the product of different stages of formation inside the rocks [12,14,15]. Rather tellingly, though, he also wrote that ‘Either these [molluscs] were terriginous [i.e. formed by plastic forces], or, if otherwise, the animals they so exactly represent have *become extinct*’ [16]. It seems that his mind was therefore open to the hypothesis shown in figure 3*c*.

By the eighteenth century, plastic forces were giving way to biblical narratives [12], and the remains of Noah's Flood were therefore being pursued by leading ‘diluvial’ geologists, such as William Buckland, well into the nineteenth century [11,12]. The ever-growing demand for mineral resources during the industrial revolution, however, led towards the discovery of an increasingly thick pile of ancient sediments, punctuated by more changes in sea level than expected from the diluvial theory [22].

There then followed a succession of fashionable null hypotheses for the earliest signs of life. The laying of Atlantic cables from 1856 onward led towards an obsession with deep sea life and hence with the antiquity of foraminiferid protozoans [35]. Such a hypothesis was seemingly confirmed by reports of complex, foram-like structures called*Eozoon*from the Precambrian of Canada. Although *Eozoon* was hailed by Darwin [23] as an early fossil, it was later shown to be abiogenic, formed by self-organization during metamorphic mineral growth [26]. Lister's dilemma had returned.

**Figure 4.**
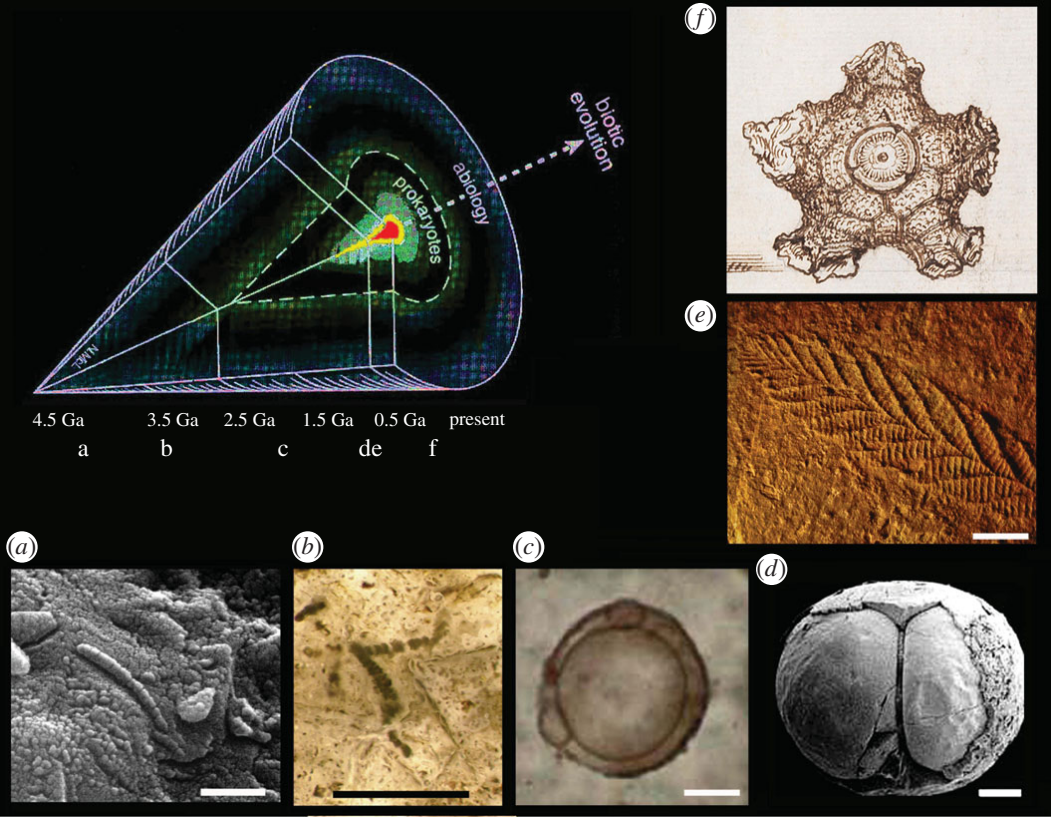
A conceptual framework for the critical testing of early fossil claims may follow this ‘cone of contention’ structure, as suggested elsewhere [39]. Fossils expand in abundance as the fossil record proceeds and as more complex forms emerge. The favoured null hypothesis may likewise shift with time, with (*a*–*e*) illustrating some ‘controversial’ fossil candidates of progressively younger age. (*a*) Approximately 4.0 Ga prokaryote-like structures from Mars (ALH 84001) [2] that were challenged by an abiogenic null hypothesis (e.g. [3]); (*b*) 3.46 Ga *Archaeoscillatoriopsis disciformis* from the Apex chert, at first compared with prokaryotes [3] that were later challenged by an abiogenic null hypothesis [4]; (*c*) 1.88 Ga complex microfossil *Eosphaera* from the Gunflint chert, usually regarded as of problematic affinity [40] but sometimes suggested to be a eukaryote cell colony [41]; (*d*) approximately 600 Ma complex microfossil *Megasphaera* from the Doushantuo Formation, whose stem-group metazoan (animal) embryo interpretation [42] has been challenged by a prokaryote hypothesis [43] and a protistan eukaryote hypothesis [44]; (*e*) 560 Ma complex megafossil *Charnia*, long regarded as cnidarian but whose animal affinities have been questioned [45,46]; (*f*) the Carboniferous crinoid whose biogenicity was at first considered moot by Lister [1] but is now interpreted as the remains of an extinct echinoderm. Scale bar is 1 μm (*a*), 40 μm (*b*), 10 μm (*c*,*d*), 30 mm (*e*).

A cyanobacteria-centred hypothesis followed later, including the global search for Precambrian stromatolites, and cyanobacteria-like microfossils (figure 4*b*) [26,40,47,48]. The search for life on Mars during the Viking expeditions of 1976 (e.g. [6]) was arguably influenced by such a ‘cyanosphere’ perspective.

There then came a major twist, following high-profile debates about bacteria-like signals and ‘Martian microfossils’ within meteorite ALH 84001 (figure 4*a*) [2,3]. A major concern of the critics was that bacteria-like mineral growths can also be generated abiogenically, within populations of complex self-organizing physico-chemical systems [5,49,50]. It has further emerged that complex stromatolite fabrics can likewise result from physical processes [48,51]. More crucially, some of the earliest candidate microfossils—once compared with cyanobacteria [3,47]—are now interpreted as abiogenic mineral growths [4,5,52–54]. Finally, it has been found that cyanobacteria are not a basal eubacterial group at all, but are relatively young and derived [55]. This ‘cyanosphere’ perspective is therefore extinct.

Early life science has now turned full circle, to embrace again the abiogenic null hypothesis. For biomorphs older than 3.0 Ga, it seems prudent not to accept them as biogenic until plausible abiogenic origins have been tested and falsified [4,5]. Comparable abiogenic tests likewise remain important for many younger Precambrian fossils [56,57]. Where biogenicity of early fossils is less in question, the chosen null hypothesis may then move upwards along a conceptual ‘cone of contention’ (see figure 4, adapted from [39]). Such a framework requires the rejection of prokaryote (e.g. bacterial) affinities in the first place, and then of simple eukaryote (e.g. protozoan and algal) affinities, before moving towards acceptance of the much sought-after but highly controversial category of early metazoan. Examples of current debates along these lines (figure 4*b*–*d*) [41–46] are here illustrated alongside Lister's drawing of a problematic fossil (figure 4*e*). Lister's dilemma is therefore back in favour.

### (b) A fully mapped context

The ability to tell biogenic from abiogenic structures is greatly aided by ‘geological intelligence’: the geological and mineralogical context of a candidate fossil, mapped at scales from kilometres to nanometres. Only in this way can a reliable narrative be constructed that passes along a time-line trajectory that broadly runs: 1, sediment deposition; 2, burial; 3, folding and metamorphism; 4, uplift and weathering. Fossils should, of course, be indigenous (initiated during phase 1) and may well show signs of modification that are distinct for each of phases 2, 3 and 4.

For Lister in 1673, the importance of context was only just emerging, and few clues were available. Although he made references to fossil localities that now allow us to place them on modern geological maps, they were rather scant. Other writings of Lister [14] more clearly highlight the finding of distinct fossils at different quarries. And while we now tend to think of William Smith as the father of stratigraphy in 1815 [58], Lister had the sagacity to promote this line of thinking [15,22,32], suggesting that distinctive stratigraphic units could be mapped to reveal their continuity across a country: ‘if it were noted how far these (Chaulk, Flint, Pyrites, Sandstone, Turf, Coal, etc.) extended, and the limits of each Soil appeared upon a Map, something more might be comprehended from the whole…than I can possibly foresee, which would make such a labour very well worth the pains’ [15].

Geological context is now fundamental for testing for all early fossil claims. It not only allows candidate fossils to be placed on a time line but also tests whether the conditions of formation were likely to have been conducive to life. Recent examples of this approach include geological mapping at the kilometre scale of the ‘questionable microfossils’ from the 3.46 Ga Apex chert [4,52–54,62] as well as new candidate fossils from the 3.43 Ga Strelley Pool arenite [61,63], accompanied by petrographic and geochemical mapping of fabrics down to the nanometre scale. Without such evidence for a viable context plus an indigenous origin, the abiogenic null hypothesis cannot be falsified convincingly.

### (c) A well-defined morphospace

Biological structures tend to occur in populations that show distinct and measurable rules for growth. These can be mapped, quantified, mathematically modelled and then placed onto a theoretical morphospace diagram. Such analysis will typically show that biological groups occupy distinct portions of a given morphospace.

Interestingly, the rules of growth for both living and fossil echinoderms were being observed and pondered by both Lister and Ray [1,21], including the presence of calcitic skeletons and pentameral symmetry. Lister also pioneered the study of mollusc shell geometry [14,15]. Such work ultimately led to the computation of a ‘Raupian’ morphospace in which bivalve, gastropod and cephalopod molluscs can now be seen to occupy distinct domains [64].

Morphospace analysis has latterly become a valuable tool for the testing of problematic relationships in very ancient fossil groups, such as Ediacaran fronds [45]. There currently remains, however, a pressing need to better characterize the nature of abiogenic morphospace and to contrast this with living examples. Over recent decades, for example, it has become clear that complex biology-like structures can be generated by wholly abiotic processes, including self-organizing systems (SOSs) involving mineral growth [49]. Such abiogenic SOSs include bacteria-like filaments [50], stromatolites and wrinkle-mats [51], microscopic trace fossils [65] and sponge-like spicules [57]. One interesting conclusion, therefore, is that Martin Lister was conceptually correct. Complexity alone is not a reliable biogenic signal.

### (d) Evidence for taphonomic behaviour

Both Lister [1] and Ray [21] clearly appreciated that fossils must often be reconstructed from separated components. This means that populations, not single specimens, are often required for the reconstruction of long-extinct organisms. Lister also realized that truly biogenic remains should show something like the following taphonomic trajectory: life posture—growth—death—transport—breakage—burial—decomposition—mineral replacement and infilling. For his fossil crinoid remains (figures 1 and 2), this trajectory was easy to read. Fossil molluscs and brachiopods are less inclined to breakage, however, making their taphonomic trajectory harder to decipher. That could explain, in part, why Lister was more inclined towards an abiogenic hypothesis for his fossil molluscs.

Evidence for a taphonomic trajectory is an important criterion for current early biosphere studies. We might expect such evidence to be incomplete or lacking within structures formed abiogenically within rocks, either by plastic forces (as Lister confronted [1]) or by mineral growth, including crystals (as we still confront [59,65]). The absence of a taphonomic trajectory within controversial biomorphs from the 3.46 Ga Apex chert [5] may therefore be significant. It contrasts with clear evidence for taphonomic patterns now being found between different microfossil taxa within the 1.88 Ga Gunflint chert [60,66].

## 8. Future developments

Biosignatures are now fundamental tools for reconstructing the ways in which our habitable planet was built (‘geobiology’), and they are increasingly being explored as signals beyond the Earth (‘astrobiology’) [6,7]. Criteria for testing the biogenicity of such signals are therefore becoming ever more urgent and more rigorous, the latter made possible by significant advances in the use of high-resolution techniques (e.g. laser Raman; nano-secondary ion mass spectrometry or nanoSIMS; focused ion beam-transmission electron microscopy or FIB-TEM; time-of-flight secondary ion mass spectrometry or ToFSIMS; synchrotron) [56]. Such techniques currently allow the mapping of morphology, ultrastructure, geochemistry and isotopes at the nanometre scale [60]. The quality and density of information about ancient life, and of possible distant life, now seems destined to grow at an exponential rate.

Technical advances (including those on robotic missions) are therefore allowing us to test for an ever wider range of biological and taphonomic features within candidate ‘biomorphs’. Hence, old and new search criteria can be usefully brought together to form a structured and practical checklist, of the kind now being used by geobiologists. Of these criteria, rules 1–4 relate to issues anticipated within Lister [1] and Ray [21], while rules 5 and 6 look towards advances in nanotechnology and imaging. Adherence to criteria of this kind will hopefully help to stimulate high-resolution analysis of biology-like behaviour and of biology-like metabolic pathways in early and remote fossil candidates (e.g. [60,67]).

## 9. Conclusion

In 1673, the biological nature of fossils was a highly controversial matter. Kircher [13] had argued that they formed by means of abiogenic ‘plastic forces’ within the rock. Hooke [20] and Steno [17] suggested that they were the remains of living organisms. Martin Lister [1] was seemingly the first to explore the ways in which direct observation could help to show whether fossil remains (in this case, Carboniferous crinoids) had grown abiogenically within the limestone or whether they had once formed part of a formerly living biological population. Prophetically, he achieved this by using the earliest known example of taphonomic reasoning in a scientific paper: that fossils of biological origin should show various lines of evidence, including those for life posture, growth, death, transport, breakage, burial, decomposition and subsequent mineral replacement and infilling.

Interestingly, abiogenicity has recently returned as a core null hypothesis. Falsification of abiogenicity now lies at the very heart of scientific debates about early life and the fossil record [5]. Taphonomic observations, such as those inferred by Lister, therefore continue to inform studies of the earliest signs of life on Earth [60]. In future, should biomorph-like signals ever be obtained from the surface of Mars, or from the icy moons of Titan, Europa and Enceladus (e.g. [6,7]), then science will have good cause to reflect again upon Martin Lister and his great dilemma of 1673.

## Author profile

**Martin Brasier** passed away on 16 December 2014, shortly after this article was accepted. He was Professor of Palaeobiology at the University of Oxford where he was also a Fellow of St Edmund Hall. His research aimed at decoding the early history of life by trying to understand big transitions in the fossil record, which involved comparing recent and ancient ecosystems using innovative high resolution techniques. He worked on the earliest well-preserved fossils of cells, mapping the earliest life on land, and the development and evolution of Ediacaran to early Cambrian organisms.
